# Effect of Impact Velocity and Angle on Impact Wear Behavior of Zr-4 Alloy Cladding Tube

**DOI:** 10.3390/ma15186371

**Published:** 2022-09-14

**Authors:** Shi-Jia Yu, Yong Hu, Xin Liu, Dong-Xing Li, Li-Ping He, Jun Wang, Zhen-Bing Cai

**Affiliations:** 1 Tribology Research Institute, Key Lab of Advanced Technologies of Materials, Southwest Jiao Tong University, Chengdu 610031, China; 2 China Institute of Atomic-Energy, Beijing 102413, China; 3 School of Materials Science and Engineering, Southwest Jiaotong University, Chengdu 610031, China

**Keywords:** cladding tube, Zr-4 alloy, dynamic response, impact wear mechanism

## Abstract

In the pressurized water reactor nuclear power plant, 316L SS chips were captured by the support grid and continued to affect the Zr-4 cladding tube, causing the fuel rods to wear and perforate. In this work, a 60° acute angle cone of 316L SS was used to simulate the cyclic impact of debris on a Zr-4 alloy tube with different initial impact velocities and impact angles. Results showed that increasing the initial impact velocity will generate a wear debris accumulation layer with a wear-reducing effect, but also promote the extension and expansion of fatigue cracks, resulting in the delamination of Zr-4 alloy tubes. The inclination of the impact angle increases the energy loss. The energy loss rate of the 45° impact is as high as 69.68%, of which 78% is generated by the impact-sliding stage. The normal force is mainly responsible for the wear removal and plastic deformation of Zr-4 alloy tubes. Tangential forces cause severe cutting in Zr-4 alloys and pushes the resulting wear debris away from the contact surfaces.

## 1. Introduction

Fuel element cladding is the first and most important safety barrier to prevent the release of radioactive products into the environment in a pressurized water reactor (PWR) [1]. The loss of its integrity not only provides huge economic losses, but also leads to significant security threat. Therefore, improving the reliability of fuel and maintaining the integrity of the nuclear fuel cladding are greatly important to the operation and long-term development of nuclear power [2].

One option, 316L stainless steel has excellent plasticity, toughness, corrosion resistance, and simple processing properties; it is widely used in the manufacture of PWR reactor internal components [3], such as nuclear power pressure vessels, control rods, main pipelines, and other key components. In the nuclear fission process, the large amount of energy released and the generated neutron radiation cause certain damage to the structural materials in the reactor, affecting the mechanical properties and microstructure of the structural materials. In addition, the boron ions, lithium ions, and chloride ions in the coolant accelerate the corrosion and wear of structural materials under the operating conditions of the reactor [4,5]. The particle size of the solid suspended particles generated in the reactor is more than 3 μm. When these particles accumulate excessively, they block the core flow passage and cause safety accidents. Accident investigation reports at home and abroad show that debris-induced fretting wear, as the second largest cause of PWR fuel rod failure, is likely to cause early damage to fuel rods [6]. Studies have shown that increasing the gap between the fuel rod and the grid induces a larger impact load between the grid and the fuel rod under the action of flow-induced vibration, resulting in severe grid-to-rod fretting wear [7,8]. However, the debris can be easily captured by the grid by reducing the gap between the fuel rod and the grid, and the cladding tube is constantly rubbed and collided under the action of water flow and pressure, causing the fuel rod to be eroded and even perforated until it leaks. In response to this phenomenon, major institutions have taken measures, such as special coatings [9], using water-lubricated drawing rod technology [10], and developing foreign matter filtering devices [11]. This approach can effectively increase the wear resistance of fuel rods, reduce the number of wear debris, and reduce the size of wear debris. However, it still cannot prevent the occurrence of foreign body abrasion. Therefore, systematically studying the failure mechanism of debris-induced fretting wear of cladding tubes is necessary.

Zr-4 alloy also has the advantages of low thermal neutron absorption cross-section, good high temperature water corrosion resistance, and sufficient comprehensive mechanical properties [12]; it is widely used as the cladding material of PWR. At present, a large number of studies on the wear mechanism of Zr-4 alloys, mainly in the background of grid-to-rod fretting (GTRF) wear, have been carried out. For example, factors, such as the flow of cooling water [13], the magnitude of the load [14], and the magnitude of the sliding [15], affect the wear of Zr-4 alloys. In addition, Jiang et al. [16,17] studied the wear properties of Zr-4 alloy tubes before and after irradiation, and found that the friction mechanism would change from adhesive wear combined with abrasive wear to abrasive wear combined with fatigue brittle fracture, and the fracture of wear debris after irradiation and spalling aggravate the wear of Zr-4 alloy. Tang et al. [18] showed that the oxide film formed after oxidation can effectively reduce the wear depth and friction coefficient of Zr-4 alloy cladding. The general direction of the cooling water is parallel to the fuel rods. The flow and vibration of the cooling water promote the fretting wear of the fuel rods and the grids, and the debris accumulated on the grids impacts the cladding tube in a lateral or inclined direction to the fuel rods. However, the research on the impact wear mechanism of 316L stainless steel on Zr-4 alloy tubes is very limited. More importantly, the effects of different initial impact velocities and impact angles on the wear behavior and wear mechanism of Zr-4 tubes are still unclear. Although some foundations for the research on the wear properties of Zr-4 alloys are available, reports on the wear mechanism of debris-induced fretting wear in Zr-4 alloy tube foreign body abrasion are few. Carrying out systematic failure mechanism research on debris-induced fretting wear of cladding tubes is necessary and valuable.

Therefore, this study mainly investigated the dynamic response and damage behavior of Zr-4 alloy tube subjected to different impact velocities and impact angles of 316L SS chips. This research is of great significance for exploring the mechanism of fretting wear caused by debris-induced fretting wear from 316L SS debris in Zr-4 alloy tubes.

## 2. Materials and Experiment

### 2.1. Materials

In this study, the size of Ø10.00 mm and thickness of 730 μm of Zircaloy-4 alloy cladding tube was cut into a length of 20 mm by wire electrical discharge machining (WEDM). The chemical composition of the Zircaloy-4 alloy (wt.%) is shown in Table 1.

The impact head was a 60° acute angle conical block with a size of Φ 10 mm × 28.66 mm; it was made of 316L stainless steel. The chemical composition of the 316L stainless steel (wt.%) is shown in Table 2. All test materials are supported by the China Institute of Atomic Energy (CIAE). Prior to the test, the samples were ultrasonically cleaned with banana water and absolute ethanol and then dried with hot compressed air.

Prior to the test, the hardness and elastic modulus of Zr-4 were measured by nano-indention. The circumferential compression test of the Zr-4 alloy pipe with 50% deformation was carried out using the universal strength testing machine (Bruker Contour GT-K1), and the load displacement curve of the material was obtained. The maximum load for the test was 50 mN, the Young’s modulus of the indenter was 1141 GPa, and the blunt radius of the indenter was 100 nm. The indentation depth of Zr-4 was approximately 817 nm, and the corresponding plastic depth was approximately 735 nm. The hardness and elastic modulus of Zr-4 are 3.32 and 99.56 Gpa, respectively. The load at the yield point of Zr-4 alloy tubes is 1062.50 N, and the deformation at this time is approximately 4%. Zr-4 alloy tubes have good compression resistance. These values indicated that Zr-4 possesses a good combination plasticity and hardness properties.

### 2.2. Experimental Equipment Parameters

**Figure 1:**
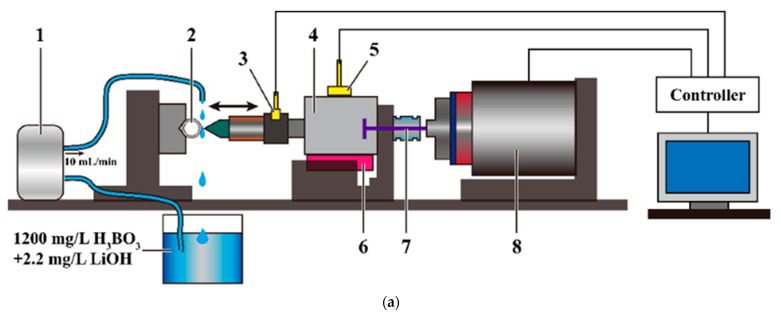

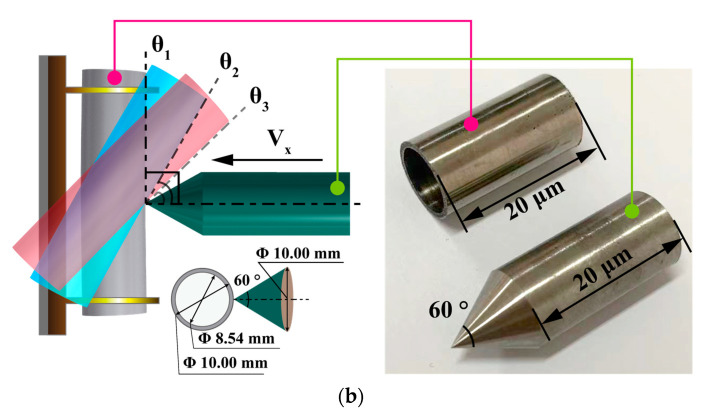
Schematic of self-developed impact wear tester (**a**): (1) water pump; (2) sample; (3) force sensor; (4) mass block; (5) displacement sensor; (6) liner guide; (7) tie rod; (8) voice coil motor, and schematic diagram of motion (**b**).

This impact wear test used a self-developed impact wear tester with controllable kinetic energy, as shown in Figure 1a. The contact force and impact velocity were recorded in real time with force sensors and displacement sensors. Based on the change in velocity, the kinetic energy theorem $E=\frac{1}{2}mv^{2}$ and the integral of velocity versus time s=∫vdt can be used to deduce the change in kinetic energy and the relative deformation of the impact process. After further analyzing the contact force, displacement, velocity and other effective data recorded by the testing machine during the test, the response and evolution of impact force, relative deformation, velocity, and energy during the testing process can be obtained.

It can realize different impact velocities by changing the speed of the voice coil motor; it can also realize different angle impacts by changing the angle of fixture. Figure 1b shows the diagram of motion, in view of the impact velocity/angle of 316L SS on the impact wear of Zr-4 alloy cladding tube, five initial impact velocities (*V_i_* = 50, 75, 100, 125, and 150 mm/s) of *θ* = 90° and three impact angles (*θ*_1_ = 90°, *θ*_2_ = 60°, and *θ*_3_ = 45°) of *V* = 100 mm/s were set. The solution of 1200 mg/L H_3_BO_3_ + 2.2 mg/L Li(OH) was used to wash the impact contact surface at the flow rate of 10 mL/min to simulate the flow of water in the primary loop of a pressurized water reactor. Each group of experiments was repeated three times, using an 800 g mass block, with a frequency of 5 Hz and *N* = 5 × 10^5^ cycles. After the test, the average value was obtained, and the corresponding standard deviation was calculated.

After the test, the surface morphology and profile of the wear mark were observed by optical microscope (OM, OLYMPUS-BX60M) and 3D morphology (Bruker Contour GT-K1), and the wear amount was measured and counted. The surface and cross-section micromorphology of the wear marks were observed by the scanning electron microscope (SEM, JSM 7800F), and the element distribution of the wear debris was characterized by X-ray spectroscopy (EDS, Tescan Mira 3 XH).

## 3. Results and Discussion

### 3.1. Influence of the Impact Velocity

#### 3.1.1. Dynamic Response

**Figure 2:**
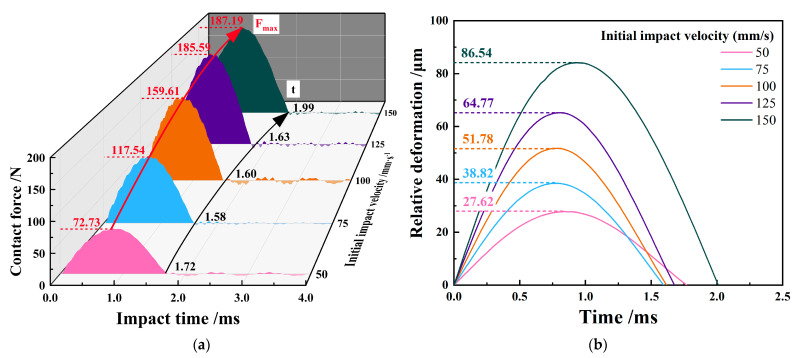
Curve of impact contact force and relative deformation with varying initial impact velocities. (**a**) Impact contact force vs. time; (**b**) relative deformation vs. time.

In the impact process, when the energy of the impact is completely transformed into elastic plastic deformation, the impact force reaches the peak. The 316L SS impact block rebounds and the impact force gradually decreases with the release of energy stored by elastic deformation [19]. The initial impact velocity *V* = 100 mm/s is approximately 120% higher than the contact peak force of *V* = 50 mm/s, and the initial impact velocity *V* = 150 mm/s is approximately 17% higher than the peak force of *V* = 100 mm/s, as shown in Figure 2a. With the increase in the initial impact velocity, the impact contact force gradually increases, and the increasing trend is gradually gentle.

Figure 2b shows that when the initial impact speed increases from *V* = 50 mm/s to *V* = 100 mm/s, the contact time between the 316L SS impact block and the Zr-4 alloy tube becomes shorter due to the increase in impact speed. However, when the initial impact speed continues to increase, the high contact stress causes severe plastic deformation of the material, and the relative deformation of the 316L SS impact block and Zr-4 alloy pipe increases greatly, resulting in a longer impact contact time [20].

**Figure 3:**
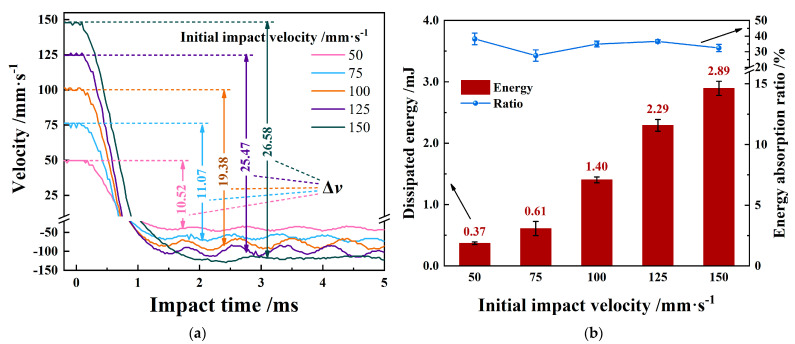
Dynamic response of velocity and dissipated energy with varying initial impact velocities. (**a**) Impact velocity response vs. time; (**b**) absorbed energy evolution.

Figure 3 shows the dynamic response of velocity and energy when the initial impact velocity is increased. As shown in Figure 3a, with the increase in the initial impact speed, the difference (Δ*V*) between the rebound speed (*V*_2_) and the initial impact speed (*V*_1_) becomes larger. 

Figure 3b shows the response of energy dissipation calculated by kinetic energy theorem $E=\frac{1}{2}mv_{1}{}^{2}-\frac{1}{2}mv_{2}{}^{2}$. The increase in initial impact velocity dissipates more energy, but its energy absorption rate is always maintained in the range of 30–40%. The Zr-4 alloy tube mainly undergoes elastic deformation during impact, and only produces evident elastic plastic deformation in the impact contact area due to the high hardness and high strength of Zr-4 alloy material. Therefore, more energy is dissipated, indicating greater plastic damage in the impact contact area.

#### 3.1.2. Wear Morphological Analysis

**Figure 4:**
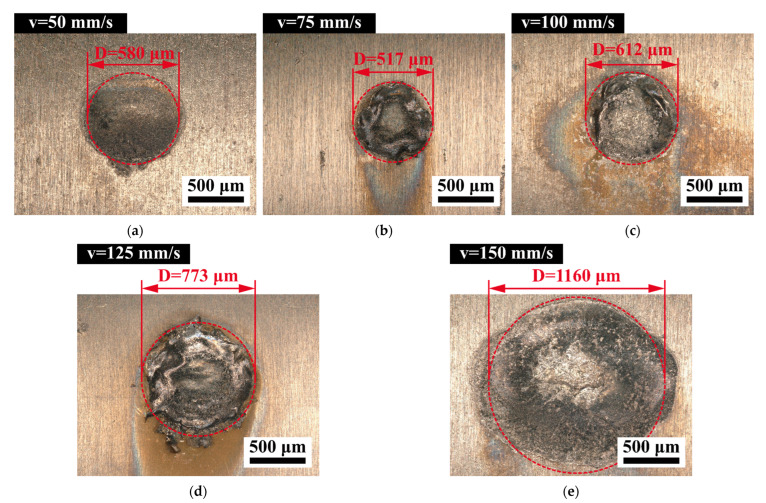

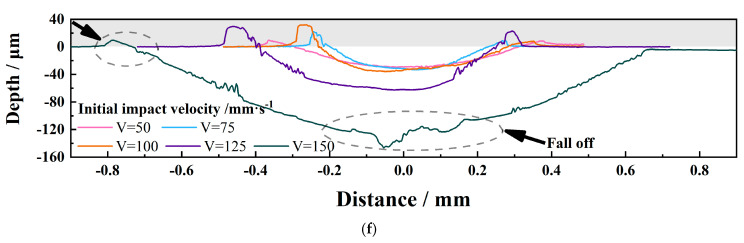
OM images and profile micrographs of the worn surfaces with varying initial impact velocities. (**a**) *V* = 50 mm/s, (**b**) *V* = 75 mm/s, (**c**) *V* = 100 mm/s, (**d**) *V* = 125 mm/s, (**e**) *V* = 150 mm/s, and (**f**) sectional profiles of worn scars under varying impact velocities.

In the previous section, we analyzed the data collected by the self-developed impact wear tester to explore the change of the impact process response in 316L SS and Zr-4 alloy tubes with the increase of the initial impact speed. The worn scars of Zr-4 alloy tubes were analyzed and discussed to further reveal the damage behavior and wear mechanism of Zr-4 alloy tubes caused by the increase in initial impact velocity. Figure 4 shows that 316L SS cyclically impacts Zr-4 alloy tubes at different initial impact speeds, providing OM images and profile micrographs of worn scars after *N* = 5 × 10^5^. 

As shown in Figure 4a−e, with the increase in the initial impact speed, the size of the worn scar decreases slightly and then increases significantly, and some of the wear mark edges exhibit evident plastic deformation bulges. The contact area between the 316L SS and Zr-4 alloy tube is small during impact because the shape of the impact block used in the test is 60° acute angle conical, resulting in much concentrated stress. The increase in the initial impact velocity leads to the increase in the contact stress, resulting in severe plastic flow of the material at the edge of the worn scar.

As shown in the cross-sectional profile of the worn scar in Figure 4f, with the increase in the initial impact velocity, the plastic flow of the material is intensified, and the material bulge at the edge of the worn scar is increased. However, when the initial impact velocity *V* = 150 mm/s, the plastic deformation bulge at the edge of the worn scar is low, but the damage at the center of the wear mark is more serious, and the profile of the worn scar at the bottom of the wear mark is rough. The simulation results of Qi et al. [21] show that during the high-speed impact process, a large amount of energy is consumed in the formation and expansion of cracks, and the proportion of energy loss caused by the plastic deformation of the material is reduced.

**Figure 5:**
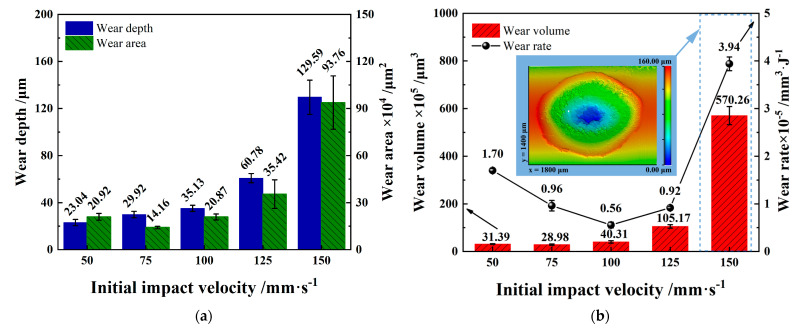
Wear statistics under varying initial impact velocities. (**a**) Wear depth and wear area; (**b**) wear volume and wear rate.

Figure 5 shows the statistics of maximum wear depth, wear area, wear volume, and wear rate under different initial impact speeds. With the increase in initial velocity and impact velocity, the maximum wear depth gradually increases, where the wear area, wear volume, and wear rate of the worn scar initially decrease slightly and then increase significantly. When the initial impact velocity is *V* = 50 mm/s, the energy dissipated by the impact is the lowest, but the wear volume and wear rate are higher than *V* = 75 mm/s, *V* = 100 mm/s, and *V* = 125 mm/s, as shown in Figure 5b.

When the initial impact velocity *V* = 150 mm/s, the wear rate increases significantly, higher than other initial impact velocities, reaching 3.94 mm^3^/J. Evidently, materials fall off at the bottom of the worn scar with initial impact velocity *V* = 150 mm/s. 

**Figure 6:**
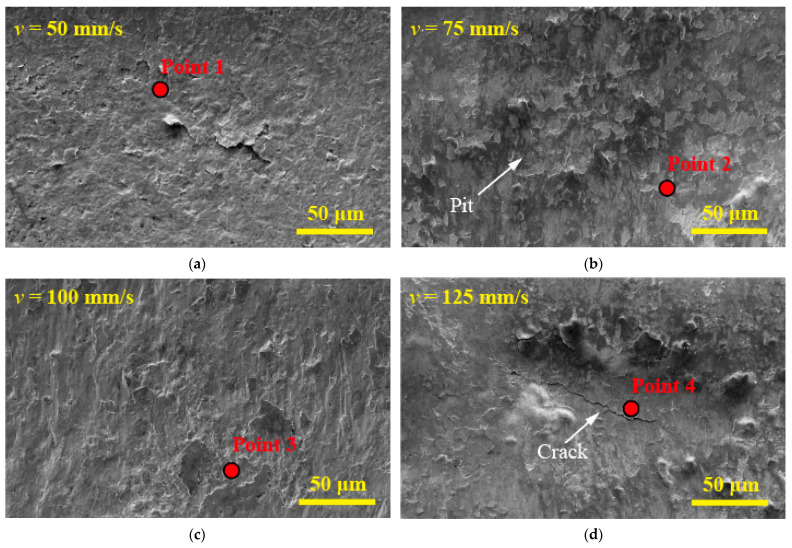

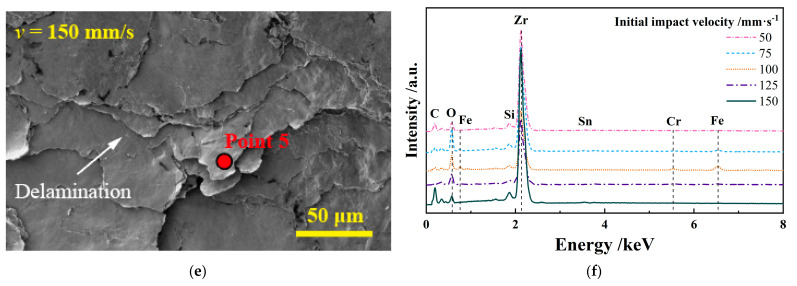
SEM morphology and EDS of wear scar center under varying initial impact velocities. (**a**) *V* = 50 mm/s, (**b**) *V* = 75 mm/s, (**c**) *V* = 100 mm/s, (**d**) *V* = 125 mm/s, (**e**) *V* = 150 mm/s, and (**f**) EDS.

The causes of these phenomena are further explored by analyzing the morphology and composition of the wear debris in the central area of the worn scar caused by different initial impact velocity. As shown in Figure 6a, the impact contact force is low when *V* = 50 mm/s, and the material damage caused by impact wear is small. In addition, the wear debris generated in the impact process leaves the contact area under the washing of lithium borate solution, resulting in only a small amount of wear particles adhering to the wear marks. The abrasive particles generated by impact adhere to the contact surface after high deformation, grinding, and oxidation when the initial impact velocity increases to *V* = 75 and 100 mm/s. As a result, a debris accumulation layer, which reduces wear [22,23], is formed, as shown in Figure 6b–c. Compared with the debris accumulation layer with complete surface structure formed when the initial impact velocity *V* = 100 mm/s, there are many pits on the surface of the debris accumulation layer with the initial impact velocity *V* = 75 mm/s. The surface of the debris accumulation layer falls off under the action of repeated tensile and compressive stresses due to the weak bonding strength between wear debris and Zr-4 alloy.

In Figure 6d, when the initial impact velocity *V* = 125 mm/s, fine cracks are evident in the debris accumulation layer in the center of the worn scar. Under the normal impact force, the relative displacement of the material due to plastic deformation causes shear stress in the secondary surface layer, promoting the initiation and propagation of microcracks [24]. As shown in Figure 6e, severe delamination and fall off occur in the center area of the worn scar when the initial impact velocity *V* = 150 mm/s.

With the increase in the initial impact velocity, the formation of the debris accumulation layer increases the content of Fe, O, and Cr elements in the debris and decreases the content of Zr elements. The spalling of the debris accumulation layer decreases the content of Fe, O, and Cr elements in the debris and increases the content of Zr elements, as shown in Figure 6f and Table 3.

The Zr element in the wear debris is mainly obtained from the Zr-4 alloy tube, and the Fe and Cr elements are mainly from the 316L SS impact block. O mainly reacts with Fe in the 316L SS friction pair because Zr is difficult to oxidize at room temperature. Therefore, the content of O is positively correlated with the Fe and Cr contents and inversely correlated with the Zr contents. The formation and exfoliation of the wear debris accumulation layer is the main reason for the change in Zr, Fe, O, and Cr elements.

**Figure 7:**
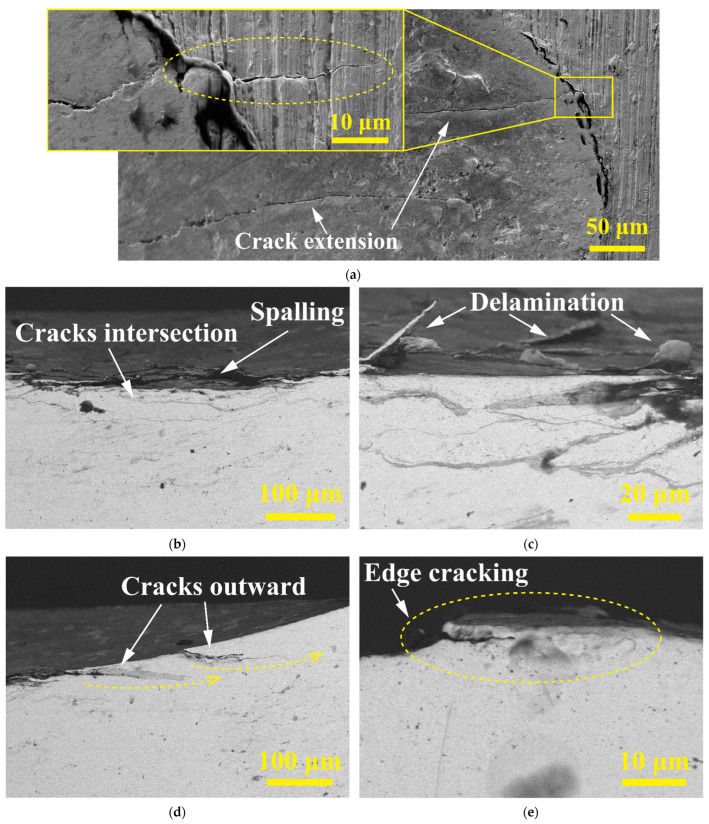
The crack distribution SEM morphology of the surface and cross-section during initial impact velocity of 150 mm/s. (**a**) Surface circumferential cracks; (**b**) cracks’ intersection; (**c**) delamination; (**d**) cracks extend outward; (**e**) plastic deformation cracks.

With the increase in the initial impact velocity, the impact contact force increases, thereby increasing the relative displacement of the material and producing more severe plastic damage. At the same time, under the action of repeated high-strength tensile and compressive stress, a large number of fatigue cracks are generated on the contact surface of the worn scar when the initial impact velocity *V* = 150 mm/s, as shown in Figure 7a. Under the action of cyclic impact force, the fatigue crack extends outward and to the noncontact substrate surface of the Zr-4 alloy tube, resulting in a larger damage area [25].

Figure 7b shows that the intersection of the intermediate regions of the cracks results in a large area of material exfoliation. The central area of the wear scar in Figure 7c caused evident delamination and spalling [26]. The outward expansion of the crack as shown in Figure 7d and the plastic deformation crack at the edge of the worn scar as shown in Figure 7e, promotes the peeling of the plastic deformation bulges at the edge of the worn scar.

### 3.2. Influence of the Impact Angle

#### 3.2.1. Dynamic Response

When the impact angle is *θ* *=* 90°, the Zr-4 alloy tube is only subject to the force perpendicular to the contact surface. The relative displacement between 316L SS impact block and Zr-4 alloy tube mainly comes from elastic plastic deformation. When the impact angle is inclined, the impact load can be divided into normal force perpendicular to the contact interface and tangential force parallel to the contact interface. The elastic plastic deformation between the 316L SS impact block and Zr-4 alloy tube caused relative displacement, and tangential force caused sliding of the contact surface.

**Figure 8:**
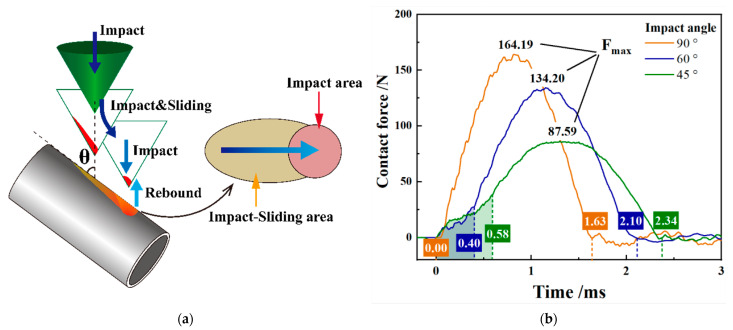
Wear statistics of the worn area under varying initial impact velocities. (**a**) Impact velocity response vs. time; (**b**) impact contact force vs. time.

As shown in Figure 8a, the impact process of the inclined angle is divided into two stages: the impact-sliding stage and the impact stage. The damage to the Zr-4 alloy pipe corresponds to the impact-sliding area and the impact area [27]. In Figure 8b, the impact contact force only increases slightly in the impact-sliding stage but increases greatly and peaks in the impact stage. The research shows that the lower initial impact velocity indicates smaller peak value of the contact force generated.

**Figure 9:**
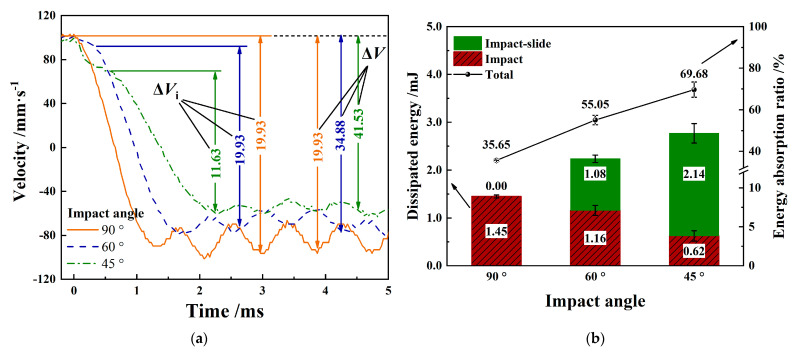
Energy response with varying impact angle. (**a**) Energy response vs. time; (**b**) dissipated energy evolution.

Figure 9a shows that the presence of the impact-sliding stage reduced the initial impact velocity of the impact stage to lower than *V* = 100 mm/s. The contact peak force (*F_max_*) of *θ* = 60° and *θ* = 45° impact decreases from 164.19 N to 134.20 N and 87.59 N, respectively. In addition, with the inclination of the impact angle, the time of impact sliding stage becomes longer, and the contact time between the 316L SS impact block and the Zr-4 alloy tube becomes longer.

The energy response curve of Figure 9a can be obtained using the kinetic energy theorem. Through the energy at the time of contact *E*_0_, the energy at the time of initial impact *E*_1_ and at the time of rebound separation *E*_2_, the energy dissipated at the impact-sliding stage, and the impact stage at three different angles can be obtained. 

As shown in Figure 9b, with the inclination of the impact angle, the energy consumption in the impact-sliding stage gradually increases, the energy consumption in the impact stage gradually decreases, and the impact-sliding stage becomes the main stage of energy dissipation. At *θ* = 45° impact, the energy dissipation in the impact-sliding stage accounts for 78% of the total energy dissipation. Moreover, the energy absorption rate gradually increases with the inclination of the impact angle. When the impact angle is *θ* = 45°, the total energy loss is as high as approximately 70%. More energy dissipation indicates greater damage of the material.

#### 3.2.2. Wear Morphological Analysis

**Figure 10:**
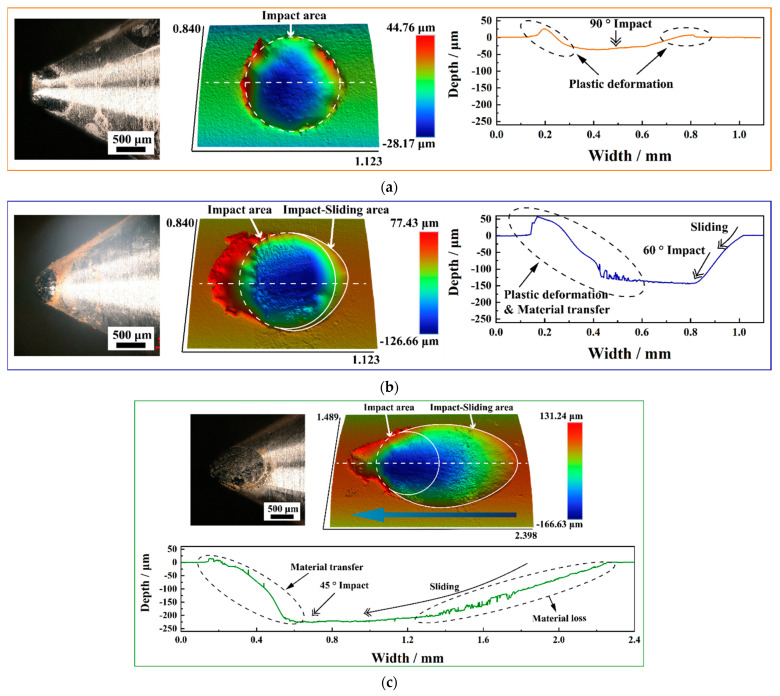
Wear of impact blocks and tube specimens at different impact angles. (**a**) *θ* = 90°; (**b**) *θ* = 60°; (**c**) *θ* = 45°.

Figure 10 shows the wear morphology of impact block 316L SS and the 3D topography and sectional profiles of worn scars of Zr-4 alloy tube under different impact angles. The damage area of the acute angle cone impact block increases with the inclination of the impact angle, and the damage changes from complete top wear to side wear.

Correspondingly, the morphology of the worn scars on the Zr-4 alloy tubes changed from a circular impact area formed only by the impact stage to a composite area of an elliptical impact sliding area formed by the impact-sliding stage and a circular impact region formed by the impact stage; the length of the worn scars increased [28]. The existence of the impact-sliding stage reduces the initial impact velocity entering the impact stage with the inclination of the impact angle; thus, the size of the circular impact area decreases. As shown in the 3D topography in Figure 10, the maximum wear depth of the worn scars of *θ* = 90°, *θ* = 60°, and *θ* = 45° impact is located in the circular impact area.

Figure 10a shows high bulges around the *θ* = 90° impact worn scar; however, in Figure 10b–c, the protrusions of *θ* = 60° and θ = 45° impact worn scar edge only occur at the edge of the circular impact area on the left, and no evident bulges are found at the edge of the elliptical impact-sliding area. Moreover, the sectional profiles of the impact-sliding area of the *θ* = 45° impact worn scar is rough, and a large amount of material wear removal is found.

**Figure 11:**
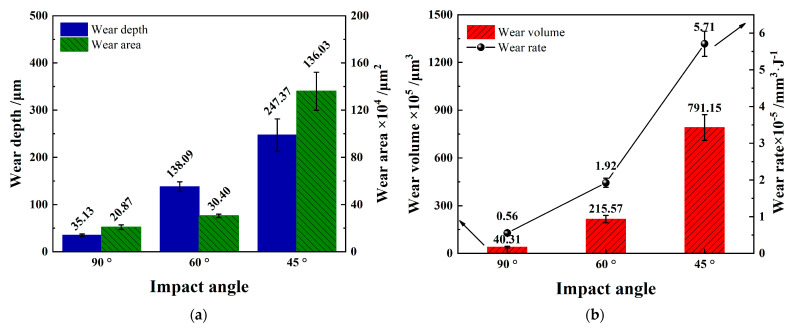
Wear statistics of the worn under varying impact angles. (**a**) Wear depth and wear area; (**b**) wear volume and wear rate.

Figure 11 shows the statistics of the maximum wear depth, wear area, wear volume, and wear rate of the Zr-4 alloy tube caused by different impact angles. With the inclination of the impact angle, the maximum wear depth, wear area, wear volume, and wear rate increase, and the wear rate of *θ* = 45° impact with the most energy loss in the impact-sliding stage is much higher than *θ* = 90° and *θ* = 60°. This phenomenon shows that the impact-sliding stage is more likely to cause material damage than the impact stage.

**Figure 12:**
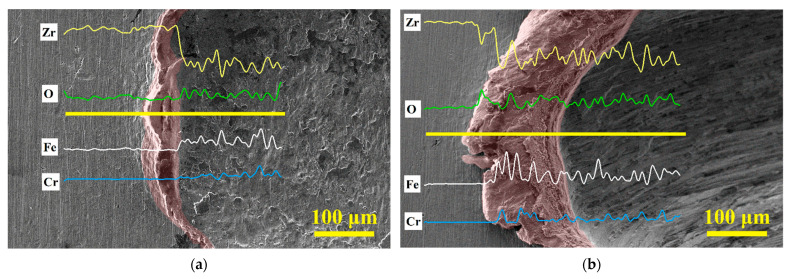

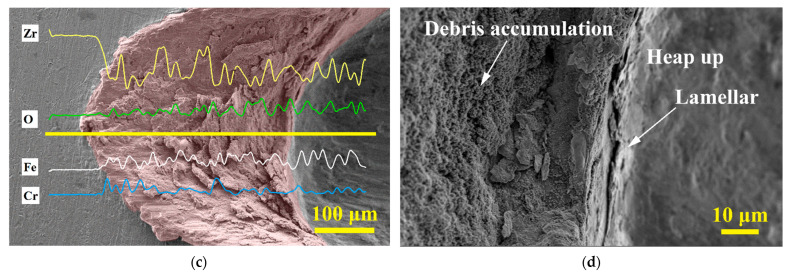
Material accumulation on the left side under different impact angles. (**a**) *θ* = 90°; (**b**) *θ* = 60°; (**c**) *θ* = 45°; and (**d**) wear debris accumulation.

Material accumulation is observed on the left side of the impact area at different impact angles, and the area of material accumulation increases with the inclination of the impact angle. The line scan data in Figure 12a show that the material accumulation on the left side of *θ* = 90° impact is the plastic deformation of Zr-4 alloy matrix, and the wear debris is accumulated on the impact contact surface. However, the material accumulation on the left side of the impact at *θ* = 60° and *θ* = 45° represents a large amount of wear debris generated during the wear process. The wear debris accumulation at *θ* = 45° impact is more serious than that at *θ* = 60° impact, as shown in Figure 12b–c. The debris are stacked into lamellar under the joint action of the normal impact force and the tangential force as shown in Figure 12d.

**Figure 13:**
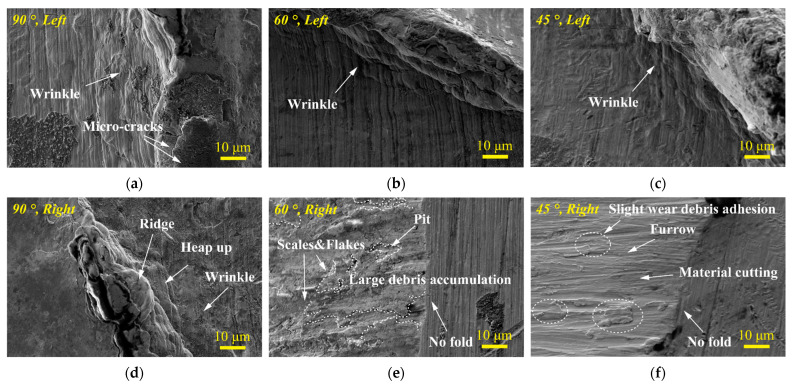
SEM morphology of wear scar edges under varying impact angles. (**a**) *θ* *=* 90°, left; (**b**) *θ* *=* 60°, left; (**c**) *θ* *=* 45°, left; (**d**) *θ* *=* 90°, right; (**e**) *θ* *=* 60°, right; (**f**) *θ* *=* 45°, right.

In Figure 13, the morphologies of the left and right edges of worn scars at different impact angles are compared. Generally, when the impact angle is *θ* = 90°, *θ* = 60°, and *θ* = 45°, the left side of the worn scar is the impact area, which promotes the plastic flow of the material under the action of the high contact force at the impact stage. Therefore, when the impact angle is *θ* = 90°, *θ* = 60°, and *θ* = 45°, folds are observed on the left edge of the worn scar, as shown in Figure 13a–c.

Figure 13d−f shows that only when the impact angle is *θ=* 90°, wrinkles and evident plastic deformation bulges are observed, whereas when the impact angle is *θ* *=* 60°and *θ* *=* 45°, the edge of the right impact-sliding area of the worn scar has no fold.

**Figure 14:**
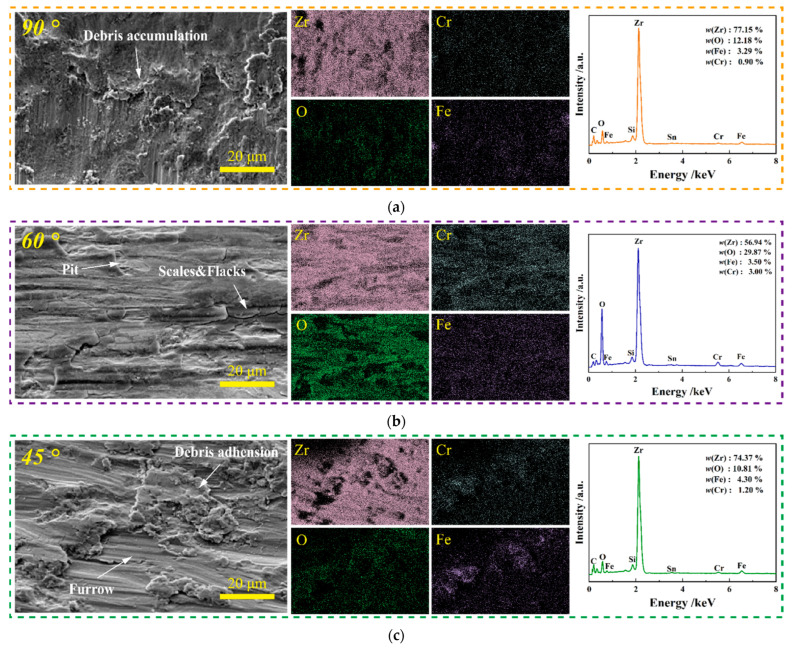
SEM morphology and element distribution of wear debris on the surface of wear scar at different impact angles. (**a**) *θ* = 90°; (**b**) *θ* = 60°; (**c**) *θ* = 45°.

The wear mechanism of Zr-4 alloy tubes under different impact angles is further analyzed by combining the morphology and composition of wear debris on the surface of each worn scar in Figure 14. With the inclination of the impact angle, the cutting of the material by the tangential force causes the damage form of the material to become mainly wear removal [29], and more wear debris are generated while increasing the contact area. A large amount of wear debris generated by the tangential force is accumulated on the contact surface in sheets under the action of the normal impact force because the contact force at the impact angle of *θ* *=* 60° is still dominated by the normal impact force, as shown in Figure 13e.

Figure 14a,b shows that, compared with *θ=* 90° impact, the content of Zr elements on the surface of wear marks under *θ* *=* 60° impact decreases, and the content of Fe, O, and Cr elements increases, thereby verifying the large number of debris deposits on the surface of worn scars under *θ* *=* 60° impact. When the impact angle is *θ* *=* 45°, the contact force is mainly tangential force. At this time, the plastic deformation manifests as cutting, and evident furrows and microcracks appear on the surface of the wear scar. Under the action of external force, these cracks propagate along the surface, resulting in the peeling off of the adhering wear debris on the contact surface, resulting in more serious material damage [30]. However, a large amount of wear debris is pushed away from the contact surface, and only a small amount remains in the impact-sliding area. Therefore, compared with *θ* *=* 60° impact, the contents of Fe, O, and Cr in *θ* *=* 45° impact evidently decreased, as shown in Figure 14c.

### 3.3. Impact Wear Mechanism

**Figure 15:**
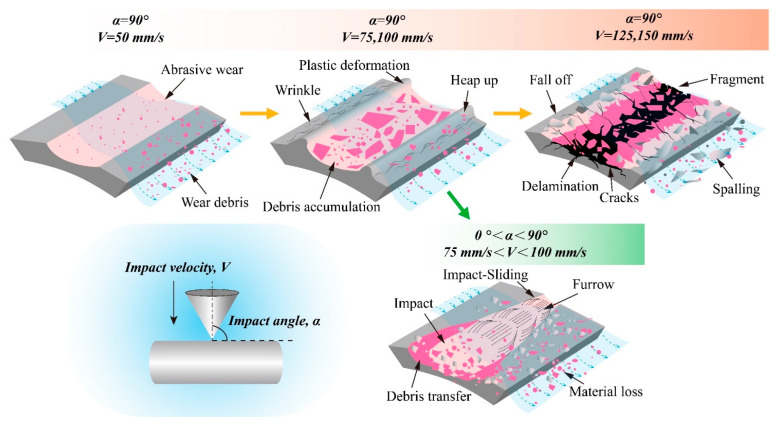
Schematic of the wear mechanisms.

Through the analysis and discussion of the test results of different initial impact velocities and impact angles, the wear mechanism of Zr-4 alloy tube is shown in Figure 15. During low-speed impact, a small amount of wear debris is washed away from the contact surface, and the impact block and Zr-4 alloy pipe mainly undergo two body abrasive wear. The impact damage area and volume of Zr-4 alloy tubes are large, but the wear depth is low [31].

With the increase in the initial impact velocity, the wear mechanism of the Zr-4 alloy tube is mainly plastic flow and spalling. At this time, the plastic deformation at the edge of the wear mark is intensified, and the wear debris accumulation layer with an antiwear effect is formed in the contact area of the worn scar. With the increase in initial impact velocity, the extension and propagation of fatigue crack caused by repeated tensile and compressive stress become the main wear mechanism [32]. The intersection of fatigue cracks in the central region leads to severe delamination, and the outward lateral propagation causes damage to the uncontacted surface of the Zr-4 alloy tube.

The inclination of impact angle leads to an impact-sliding stage prior to the impact stage, and the main mechanism of wear are peeling and cutting. Cutting causes more severe wear removal of the material and pushes the generated debris away from the contact surface. The damage degree cannot be directly determined because the damage forms of the Zr-4 alloy tube caused by the initial impact velocity and the inclined impact angle are different. However, the wear of Zr-4 alloy tube can be reduced by reducing the initial impact velocity and the tangential force [33].

## 4. Conclusions

The Zr-4 alloy tube was impacted by a 316L SS 60° acute angle conical impact block at different initial impact velocities and impact angles for *N* = 5 × 10^5^ times, and the following conclusions were reached:

(a) The inclined impact can be divided into two stages: (I) impact-sliding stage and (II) impact stage. A more inclined impact angle indicates greater energy dissipation in the impact-sliding stage and greater material wear.
(b) The increase in the initial impact velocity promotes damage of the material from spalling and plastic deformation to fatigue crack extension and propagation; although inclined impact angle can reduce the impact contact force and reduce the damage caused by impact, cutting in the impact-sliding stage causes much serious material removal.
(c) The accumulated layer of wear debris produced in the impact process can reduce the wear, and the formation and falling off of the accumulated layer of wear debris are the main reasons for the change in Zr, Fe, O, and Cr elements on the surface of worn scars.

## Figures and Tables

**Table 1:** Chemical composition of Zircaloy-4 alloy in wt.%.

Materials	Element				
	O	Cr	Fe	Zr	Sn
Zircaloy-4 alloy	0.13	0.10	0.21	98.11	1.45

**Table 2:** Chemical composition of the 316L stainless steel in wt.%.

Materials	Element								
	C	Si	Mn	S	P	Cr	Ni	Mo	Fe
316L stainless steel	0.021	0.540	1.280	0.004	0.014	17.21	12.46	2.450	66.021

**Table 3:** The chemical compositions of points in wt.%.

Element	Weight %				
	Point 1	Point 2	Point 3	Point 4	Point 5
Zr	81.61	70.41	63.52	72.32	87.70
O	11.19	19.98	15.19	16.28	6.50
Fe	0.80	2.60	10.99	2.50	0.40
Cr	0.30	0.70	2.60	1.10	0.20

## Data Availability

All data were presented in this manuscript.
